# Exploring the mechanism of 6-Methoxydihydrosanguinarine in the treatment of lung adenocarcinoma based on network pharmacology, molecular docking and experimental investigation

**DOI:** 10.1186/s12906-024-04497-z

**Published:** 2024-05-23

**Authors:** Xingyun Liu, Yanling Ren, Shuanglin Qin, Zerui Yang

**Affiliations:** 1 The Affiliated Nanhua Hospital, Hengyang Medical School, University of South China, Hengyang, 421000 China; 2 Key Specialty of Clinical Pharmacy, The First Affiliated Hospital of Guangdong Pharmaceutical University, Guangzhou, 510000 China; 3 School of Pharmacy, Xianning Medical College, Hubei University of Science and Technology, Xianning, 437000 China; 4 NMPA Key Laboratory for Technology Research and Evaluation of Pharmacovigilance, Guangdong Pharmaceutical University, Guangzhou, 510086 China

**Keywords:** 6-Methoxydihydrosanguinarine, Lung adenocarcinoma, Network pharmacology, Molecular docking

## Abstract

**Background:**

6-Methoxydihydrosanguinarine (6-MDS) has shown promising potential in fighting against a variety of malignancies. Yet, its anti‑lung adenocarcinoma (LUAD) effect and the underlying mechanism remain largely unexplored. This study sought to explore the targets and the probable mechanism of 6-MDS in LUAD through network pharmacology and experimental validation.

**Methods:**

The proliferative activity of human LUAD cell line A549 was evaluated by Cell Counting Kit-8 (CCK8) assay. LUAD related targets, potential targets of 6-MDS were obtained from databases. Venn plot analysis were performed on 6-MDS target genes and LUAD related genes to obtain potential target genes for 6-MDS treatment of LUAD. The Search Tool for the Retrieval of Interacting Genes/Proteins (STRING) database was utilized to perform a protein-protein interaction (PPI) analysis, which was then visualized by Cytoscape. The hub genes in the network were singled out by CytoHubba. Metascape was employed for GO and KEGG enrichment analyses. molecular docking was carried out using AutoDock Vina 4.2 software. Gene expression levels, overall survival of hub genes were validated by the GEPIA database. Protein expression levels, promotor methylation levels of hub genes were confirmed by the UALCAN database. Timer database was used for evaluating the association between the expression of hub genes and the abundance of infiltrating immune cells. Furthermore, correlation analysis of hub genes expression with immune subtypes of LUAD were performed by using the TISIDB database. Finally, the results of network pharmacology analysis were validated by qPCR.

**Results:**

Experiments in vitro revealed that 6-MDS significantly reduced tumor growth. A total of 33 potential targets of 6-MDS in LUAD were obtained by crossing the LUAD related targets with 6-MDS targets. Utilizing CytoHubba, a network analysis tool, the top 10 genes with the highest centrality measures were pinpointed, including MMP9, CDK1, TYMS, CCNA2, ERBB2, CHEK1, KIF11, AURKB, PLK1 and TTK. Analysis of KEGG enrichment hinted that these 10 hub genes were located in the cell cycle signaling pathway, suggesting that 6-MDS may mainly inhibit the occurrence of LUAD by affecting the cell cycle. Molecular docking analysis revealed that the binding energies between 6-MDS and the hub proteins were all higher than − 6 kcal/Mol with the exception of AURKB, indicating that the 9 targets had strong binding ability with 6-MDS.These results were corroborated through assessments of mRNA expression levels, protein expression levels, overall survival analysis, promotor methylation level, immune subtypes andimmune infiltration. Furthermore, qPCR results indicated that 6-MDS can significantly decreased the mRNA levels of CDK1, CHEK1, KIF11, PLK1 and TTK.

**Conclusions:**

According to our findings, it appears that 6-MDS could possibly serve as a promising option for the treatment of LUAD. Further investigations in live animal models are necessary to confirm its potential in fighting cancer and to delve into the mechanisms at play.

**Supplementary Information:**

The online version contains supplementary material available at 10.1186/s12906-024-04497-z.

## Introduction

Lung cancer, composed of approximately 85% non-small-cell lung cancer (NSCLC) and 15% small cell lung cancer (SCLC), is one of the most prevalent malignant cancers worldwide, with over 1.4 million deaths each year [1]. According to Global cancer statistics 2022 published in 2024, with almost 2.5 million new cases and over 1.8 million deaths worldwide, lung cancer is the leading cause of cancer morbidity and mortality in 2022 [2]. Another study estimates that there will be 3.8 million incident cases and 3.2 million deaths globally due to lung cancer in 2050 [3].To date, Lung adenocarcinoma (LUAD) is the most common subtype of NSCLC [4, 5]. Despite improvements in chemotherapy, radiotherapy, and surgery, the prognosis of NSCLC still remains poor, and the five-year survival rate is only approximately 18% [6]. Patients with advanced-stage disease are treated with chemotherapeutic medications such as platinum [7]. However, the development of resistance to chemotherapeutic medications and the occurrence of adverse responses to these treatments have become major challenges in modern oncology [8]. The mechanisms underlying resistance to chemotherapeutic medications are multifactorial. It has been reported that cisplatin resistance often occurs due to a cellular defense mechanism that confers resistance by reducing the ability to mediate apoptosis, enhancing the repair of DNA damage, altering cell cycle checkpoints, and disrupting cytoskeleton assembly [9]. Nevertheless, the exact mechanisms resistance to chemotherapeutic medications remain largely unclear [10]. Meanwhile, targeted therapy and immunotherapy have been developed to overcome these problems, but it has faced to acquired resistance, poor therapeutic response, systemic immune dysfunction [11, 12]. The clinical outcomes and effects of neoadjuvant therapy (including Chemotherapy/Radiotherapy, Targeted Therapy and Immunotherapy) for NSCLC are still controversial due to its apparent advantages and disadvantages [13]. Considering the exists controversy concerning the efficacy of current therapy for patients with NSCLC, searching for low toxicity and effective anti-cancer drugs and new drug targets has been a research direction in recent years.

Traditional Chinese medicine (TCM) has long been utilized as a complementary treatment for various types of cancer, such as lung cancer [14]. TCM is characterized by the utilization of medicines derived from natural herbs, rather than being created through chemical synthesis [15]. These herbal drugs have low toxicity and exert complex anti-cancer effects through a variety of intricate mechanisms [16]. Therefore, the active ingredients extracted from Chinese herbs have become a hot spot for global research [17]. *Macleaya cordata* (Chinese name “Bo-luo-hui”) is a perennial herb that belongs to the Papaveraceae family and is typically prescribed as a traditional antibacterial medicine, whose effect and usage were well documented in Ben-Cao-Shi-Yi, a Chinese encyclopedia of botany and medicine from the early Tang dynasty [18]. According to literature, it has significant therapeutic effects on ulcers, snake and insect bites, anti-tumor effects, and improving liver function [19]. The main component of *Macleaya cordata* extract is alkaloid. More than 70 kinds of alkaloids have been reported to be isolated and identified from *Macleaya cordata*, and 6-Methoxydihydrosanguinarine (6-MDS) belongs to one of the isoquinoline alkaloids. It is reported that 6-MDS has antimicrobial activity and has significant inhibitory activity against *Staphylococcus aureus* [20]. In addition, 6-MDS can induce proliferation and apoptosis of HT29 cells and Hep G2 cells, with IC_50_ values of 3.8 ± 0.2 and 5.0 ± 0.2 µM, respectively [21, 22]. The latest research showed that 6-MDS can induce apoptosis and autophagy of breast cancer MCF-7 cells by inhibiting PI3K/AKT/mTOR signaling pathway by accumulating ROS, which has great potential in the treatment of cancer [20]. Also, 6-MDS exhibits cytotoxicity and sensitizes TRAIL-induced apoptosis of hepatocellular carcinoma cells through ROS-mediated upregulation of DR5 [23]. However, its anti‑LUAD effect and its mechanism have not yet been reported.

Traditional studies often focus on a single gene or target, ignoring the complexity and systematicness of biological processes. However, Chinese medicine has multi-target and multi-pathway mechanisms of action for treating diseases. Therefore, it is necessary to use big data to mine all existing targets and pathways related to 6-MDS and LUAD. In recent years, the network pharmacology has developed, which integrates the system biology and pharmacology, integrates the biological network and pharmacology, changes the traditional search for a single target to comprehensive network analysis, emphasizes the interaction mode of multicomponents, multitargets, and multipathways, and is especially suitable for predicting the action target and possible mechanism of natural compounds from traditional Chinese medicines or different plants [24, 25]. In the present study, our team used the network pharmacology technique and in vitro experiment to access the molecular pathways behind 6-MDS’s ability to block LUAD proliferation.

## Methods

### Cell experiment verification

#### Cell culture

A549 cells were purchased from American Type Culture Collection (ATCC, Manassas, VA) and were cultured in RPMI 1640 medium (CT1875500BT, Gibco, ThermoFisher Scientific, USA) supplemented with 10% fetal bovine serum (164210-50, FBS, Procell, Wuhan, Hubei, China) plus penicillin G (15140-122, Gibco, ThermoFisher Scientific, USA, 100 U/mL) and streptomycin (15140-122, Gibco, ThermoFisher Scientific, USA,100 U/mL) at 37 °C under 5% CO_2_ [26].

#### Cell viability assays

Cells were inoculated into 96-well flat-bottomed microtiter plates with 6000 cells per well, after 24 h of cultivation, they were treated with 6-MDS for 24 h and 48 h, respectively. Then, on the basis of the manufacturer’s instructions, Cell Counting Kit-8 (CCK8, GK10001, GlpBio, USA) stock solution was added to each well and the plate was incubated in a cell culture incubator at 37 °C for 60 min. The absorbance at 450 nm wasmeasured using a microplate reader (Multiskan-GO, Thermo Fisher Scientific, USA) to assess the cell viability [27].

### Screening gene targets related to 6-MDS

First, the 2D, 3D structure, SDF file or SMILES of 6-MDS was downloaded from the PubChem website (https://pubchem.ncbi.nlm.nih.gov/) [28] and was then imported into the PharmMapper (Version 2017, http://www.lilab-ecust.cn/pharmmapper/) [29], SuperPred(Version 3.0, https://prediction.charite.de/) [30] SwissTargetPrediction (Version2019, http://swisstargetprediction.ch/) [31] and targetnet (Version2016, http://targetnet.scbdd.com/) [32] for predicting the target genes. The parameters of PharmMapper were set as normalized fit score > 0.1. The cut-off of SwissTargetPrediction was set as probability > 0.1. The cut-off of targetnet was set as probability > 0.001 and the cut-off of SuperPred was set as probability > 0.5. After the target genes/proteins were obtained, the protein names were transformed to the corresponding gene names through the UniProt database (Release 2024_02, http://www.uniprot.org) [33] if necessary. Finally, the target genes obtained in the above four databases were combined to form a gene set.

### LUAD-related target collection

The LUAD-related target genes were obtained in two ways. First, the differentially expressed genes (DEGs) specific to LUAD were acquired by extracting data from the GEPIA“Differential Expression Analysis” module by setting the parameter of |Log2FC| Cutoff: 1, q-value Cutoff: 0.01(http://gepia.cancer-pku.cn/detail.php?gene=&clicktag=expdiy) [34]. Secondly, other potential targets associated with LUAD were identified through exploration three public database, which were OMIM (https://www.omim.org/) [35], Drugbank (version 6.0, https://go.drugbank.com/) [36], and GeneCards databases (Version 5.20, https://www.genecards.org/) [37] utilizing the search term“lung adenocarcinoma”. Finally, potential LUAD-related targets were obtained by intersecting the DEGs extracted from the GEPIA database with the potential targets associated with LUAD sourced from the aforementioned trio of databases.

### Determining the targets of 6-MDS in LUAD

For the identification of potential target genes for 6-MDS treatment of LUAD, an analysis using Venn diagrams was conductedon 6-MDS target genes and LUAD related genes. Subsequently, shared targets were found between 6-MDS target genes and LUAD related genes, which were considered as potential targets of 6-MDS in LUAD.

### Enrichment analysis of the targets of 6-MDS in LUAD

Enrichment analysis of Gene Ontology (GO) and Kyoto Encyclopedia of Genes and Genomes (KEGG) were conducted using the online tool Metascape (Version 3.5.20240101, https://metascape.org) [38].

### Protein-protein interaction (PPI) network construction and core targets screening

The STRING database (Version 12.0, http://string-db.org/) [39], which is an online database for evaluations of PPI, was employed to delve into the relationship between the proteins encoded by the potential targets of 6-MDS in LUAD. Subsequently, the results from STRING database were exported to the plug-in CytoHubba of Cytoscape software (Version 3.9.0, https://cytoscape.org/) [40] to visualize the PPI network. Finally, maximal clique centrality (MCC) algorithm was performed to screen the top ten 10 genes, deemed as potential hub genes for subsequent investigation.

### Molecular docking

Molecular docking is mainly used for the structural docking of small molecules with target proteins, and to evaluate their binding affinity with defined binding sites. It is generally believed that the lower the energy of the ligand receptor binding conformation, the more likely this effect is to occur [41]. small molecule ligand files of chemical components were downloaded from the PubChem database, imported them into Chem3D software for the purpose of spatial structure transformation and energy optimization, and output them in mol 2 format file format. After processing with AutoDockTools 1.5.6 software, the files were saved in pdbqt format. Then the gene ID of the core target was retrieved from the Uniprot database and downloaded its corresponding PDB format file from the PDB database (http://www.rcsb.org/). After water molecule removal and ligand separation using PyMol software, the resultant macromolecular receptor file of the core target was imported into AutoDockTools 1.5.6 software for hydrogenation and saved in PDbqt format. Finally, with the help of AutoDockvina 1.1.2 software, the core target and its corresponding chemical components were molecular docked, with the binding energy serving as the docking evaluation index. The predicted value of the dissociation constant (Kd) was calculated from ΔG = RT ln(Kd) [42] where ΔG is the binding energy, R is the idea gas constant (kcal*K^− 1^ *mol^− 1^) and T is the temperature (K), with the help of an online web server developed byNovoPro Bioscience Inc. (https://www.novoprolabs.com/tools/deltag2kd).

### External validation of hub genes

#### Gene expression anaysis of hub genes

The GEPIA (http://gepia.cancer-pku.cn/) “Expression on Box Plots” and “Pathological Stage Plot” module wereemployedto explore the mRNA expression levels and pathological stages of the core targets in LUAD. A threshold of |log2FC| ≥ 1 and a significance level of *p* ≤ 0.01 were established for the analysis.

#### Protein expression analysis of core targets

UALCAN (https://ualcan.path.uab.edu/) [43] provides protein expression analysis option using data from Clinical Proteomic Tumor Analysis Consortium (CPTAC) and the International Cancer Proteogenome Consortium (ICPC) datasets [44]. Here, this database wasutilizedto conduct a comparative analysis of the protein expression levels of the hub genes inLUAD tissues and their corresponding normal tissues.

#### Overall survival analysis of hub genes

Using the GEPIA database, the potential relationship between expression of the 9 hub genes and the OS of LUAD patients was evaluated by setting *p* < 0.05 as the criteria.

#### DNA methylation level of hub genes

The UALCAN databases was used to compared DNA methylation level of hub genes between normal and LUAD tissues.

#### Correlation analysis of hub genes expression with immune cell infiltration

the online database Timer (Version 2.0, http://timer.cistrome.org/) [45] was ultized to analyze the correlation between the hub genes expression andinfiltration score of B cell, CD4 T cell, CD8 T cell, neutrophil, macrophage and dentritic cell (DC).

#### Correlation analysis of core targets expression with immune and molecular subtypes of LUAD

The TISIDB database (http://cis.hku.hk/TISIDB/index.php) is an online integrated repository portal collecting abundant human cancer datasets sourcedfrom the TCGA database [46]. The association between the expression level of core targets and immune or molecular subtypes of LAUD were assessed through the TISIDB database. Results were deemed statistically significant if the *P*-value was less than 0.05.

### Real-time PCR validation

#### Primers and chemicals

Primers were purchased from sangon biotech(Shanghai, China). 6-MDS (WP23101106) was purchased from Sichuan Weikeqi Biological Technology co. Ltd (Chengdu, Sichuan, China).

#### Real-time PCR

By using a SteadyPure Universal RNA Extraction Kit (AG21017, Accurate Biology, Hunan, China), total RNA of the cells was extracted. Subsequently, 1 µg of RNA was subjected to the reverse transcription process within a 20 µL reaction volume utilizing Evo M-MLVRTMix Kit with gDNA Clean for qPCR Ver.2 (AG11728, Accurate Biology, Hunan, China). The resulting cDNA was then employed for real-time PCR assay by using the SYBR Green Pro Taq HS qPCR Kit (AG11701,Accurate Biology, Hunan, China). The specificprimer sequences used in this study were as follows:

GAPDH-F: AAGGTGAAGGTCGGAGTCAA.

GAPDH-R: AATGAAGGGGTCATTGATGG.

CDK1-F: TAGGCGGGATCTACCATACCC.

CDK1-R: TCATGGCTACCACTTGACCTG.

CHEK1-F: CCAGATGCTCAGAGATTCTTCCA.

CHEK1-R: TGTTCAACAAACGCTCACGATTA.

KIF11-F: CGGAAAGCTAACGCCCACTCAG.

KIF11-R: TCTTATCAGCCAGTCCTCCAGTTCG.

PLK1-F: CACCAGCACGTCGTAGGATTC.

PLK1-R: CCGTAGGTAGTATCGGGCCTC.

TTK-F: AAACAGTGTTCCGCTAAGTGATG.

TTK-R: AGGGCAATTTCCAGCATTTCTA.

### Statistical analysis

GraphPad Prism 5.0 (GraphPad Software, San Diego, CA, USA; RRID: SCR_002798) was applied to analyzed the data. The analysis results of the data were presented in mean ± SD. The statistical difference between two samples was analyzed by Students t test. * means *P* < 0.05, ** means *P* < 0.01 and *** means *P* < 0.001. *P* less than 0.05 indicates statistical difference in results.

## Results

### 6-MDS inhibits proliferation of A459 cells

**Fig. 1:**
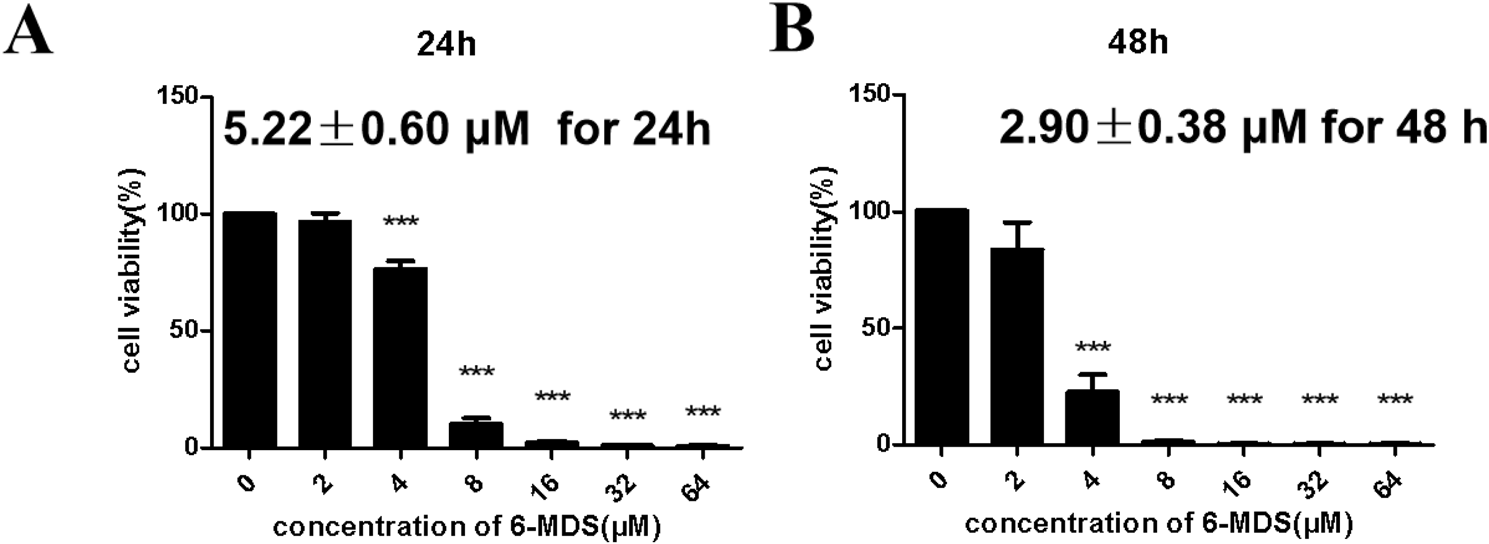
Effect of 6-MDS on the viability of A549 for 24 h **(A)** and 48 h **(B)**, respectively

To verify the anti-proliferative effect of 6-MDS on LUAD, CCK8 was used to determine the cell viability after treatment with 6-MDS for 24 h and 48 h, respectively. Gradually elevating the concentrations of 6-MDS from 2 to 64 µg/mL resulted in a dose-dependent decrease in the survival rates of LUAD cells (Fig. 1A-B), indicating a pronounced inhibitory influence of 6-MDS on LUAD cell proliferation. Specifically, 6-MDS inhibited the growth of A549 cells with an IC_50_ of 5.22 ± 0.60 µM for 24 h and 2.90 ± 0.38 µM for 48 h.

### Prediction of LUAD and 6-MDS targets

**Fig. 2:**
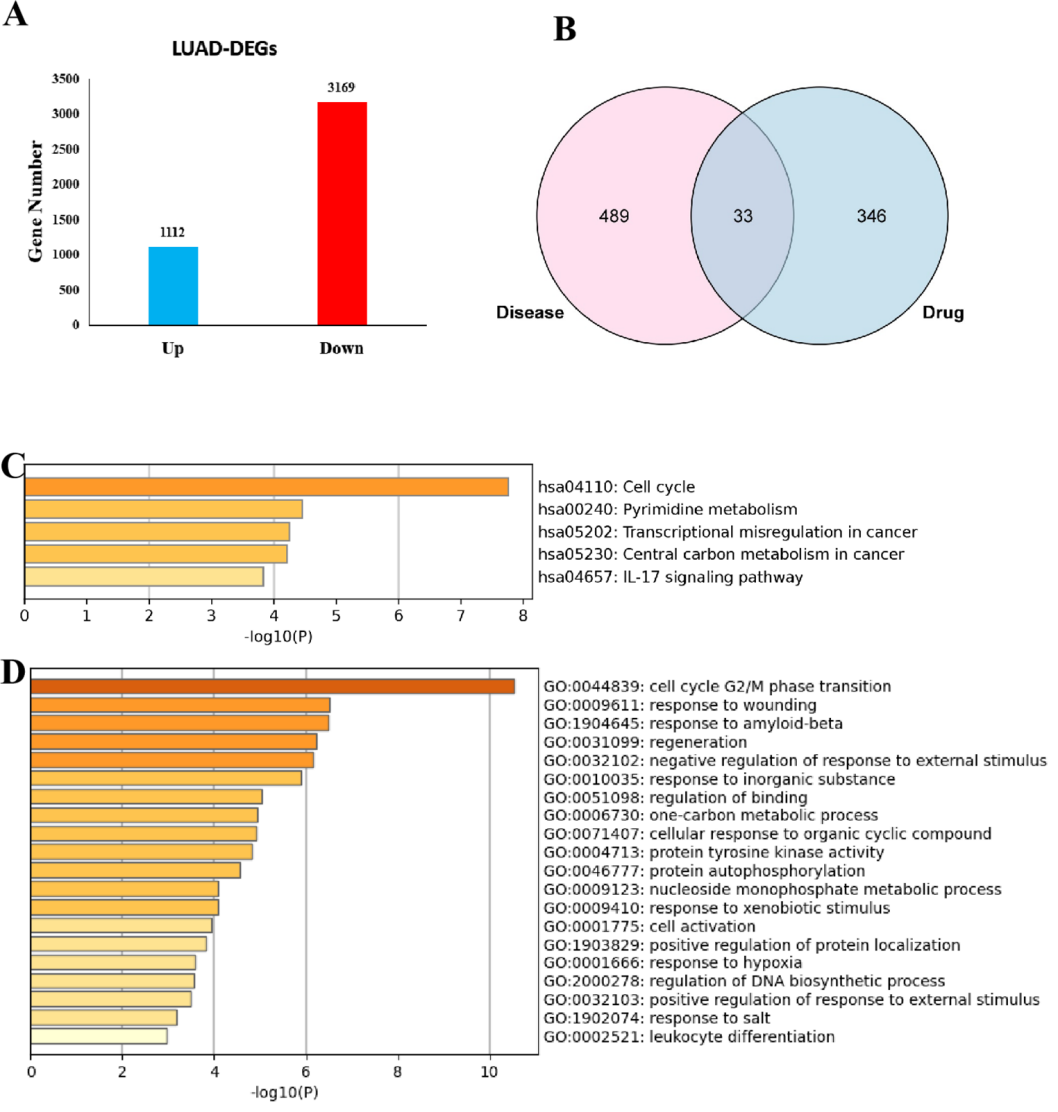
Identification of the DEGs of LUAD in the TCGA cohort and Functional enrichment analysis. **A** Box plots to visualize the DEGs; **B** Venn diagram to identify 6-MDS-related targets for LUAD. **C** KEGG enrichment analysis of the 6-MDS-related targetsfor LUAD; **D** GO enrichment analysis of the 6-MDS-related targetsfor LUAD

Using the TCGA-LUAD cohort in GEPIA database, a total of 4,246 DEGs were identified and visually displayed through box plots, including 1,112 up-regulated and 3,134 down-regulated (Fig. 2A, Table S1). Besides, 9,642 LUAD targets were extracted from the above-mentioned three database: Drugbank, GeneCards and OMIM (Table S2). Finally, 522 LUAD-related targets were obtained by Venn analysis (Table S3). As for the 6-MDS targets, a grand total of 379 target genes were secured from the PharmMapper, SuperPred, SwissTargetPrediction and targetnet (Table S4).

### Targets of 6-MDS in LUAD acquisition and functional enrichment analysis

33 potential targets of 6-MDS in LUAD were identified by crossing the LUAD-related targets with 6-MDS targets (Fig. 2B, Table S5), which were then submitted to the Metascape database to explore the potential biological functions of these genes. The results of KEGG enrichment analysis indicated that these genes were significantly enriched in cell cycle, pyrimidine metabolism as well as transcriptional mis-regulation in cancer (Fig. 2C). Regarding GO analysis, the results demonstrated that these 33 potential targets of 6-MDS in LUAD were notably related to cell cycle G2/M phase transition, response to wounding and response to amyloid-beta (Fig. 2D). These outcomes imply that 6-MDS may mainly inhibit the occurrence of LUAD by affecting the cell cycle.

### PPI network construction

**Fig. 3:**
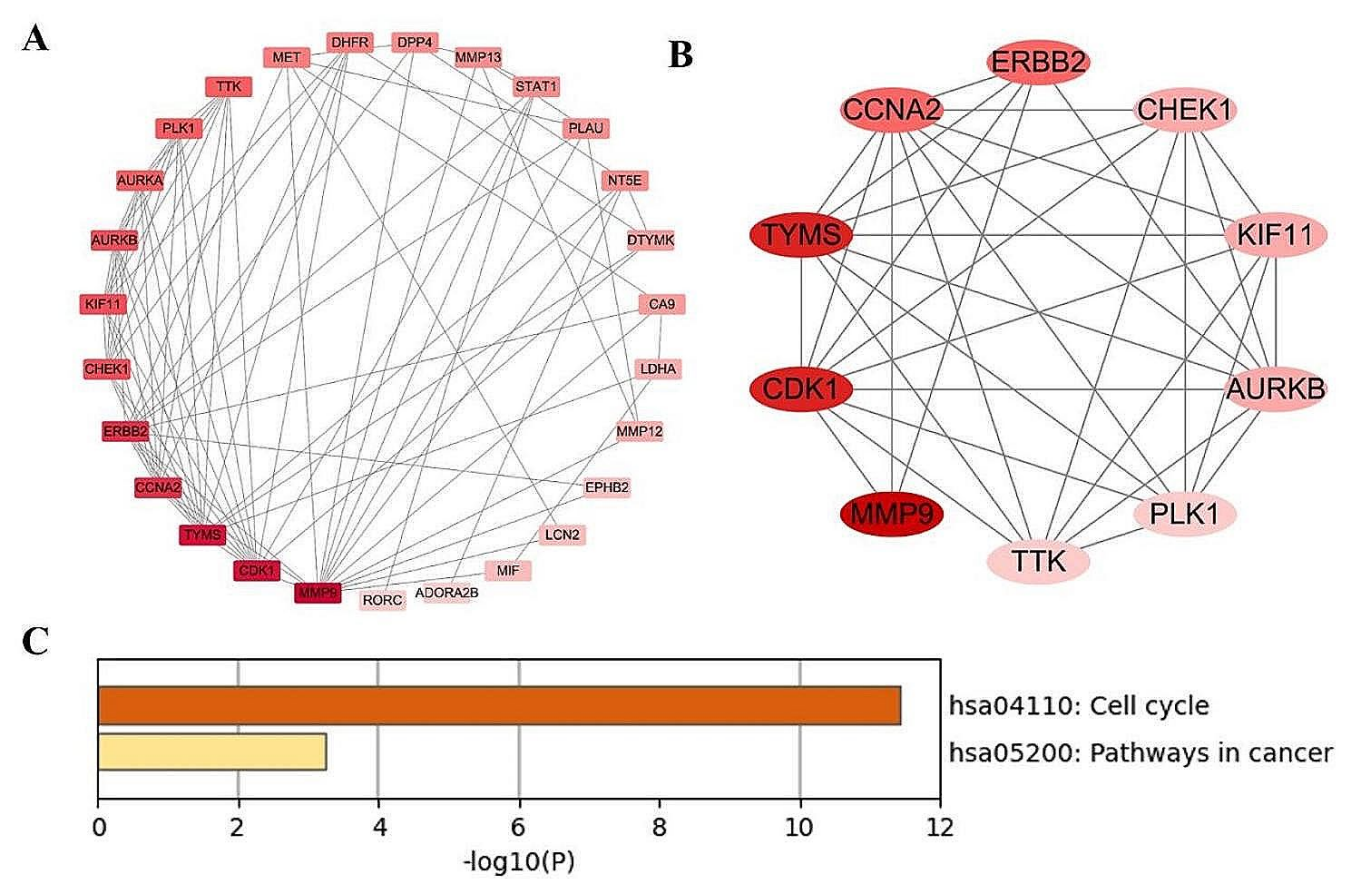
PPI network and KEGG analyses of the ten core genes. **A** PPI network visualized by Cytoscape; **B** Network of interactions of top ten hub genes; **C** KEGG enrichment analysis of the top ten hub genes

The 33 potential targets of 6-MDS in LUAD were submitted to the STRING database to obtain the PPI data. The PPI data was then visualized and analyzed using Cytoscape software (Fig. 3A). Subsequently, the cytohubba plug-in was employed to analyze the PPI network and identify the top ten hub genes, which were then displayed in Table 1; Fig. 3B. Nodes in a darker shade of red indicate higher importance. The top 10 hub genes comprised MMP9, CDK1, TYMS, CCNA2, ERBB2, CHEK1, KIF11, AURKB, PLK1 and TTK. Further analysis through KEGG enrichment analysis indicated that these hub genes were primarily involved in the cell cycle and pathways in cancer signaling pathway (Fig. 3C).

**Table 1:** 10 Hub genes identified using 4 different algorithms in the Cytohubba plug-in

Rank	Gene symbol	Full name	Degree
1	MMP9	Matrix metalloproteinase-9	14.00
2	CDK1	Cyclin-dependent kinase 1	13.00
3	TYMS	Thymidylate synthase	13.00
4	ERBB2	Receptor tyrosine-protein kinase erbB-2	11.00
5	CCNA2	Cyclin-A2	11.00
6	KIF11	Kinesin-like protein KIF11	9.00
7	AURKB	Aurora kinase B	9.00
8	CHEK1	Serine/threonine-protein kinase Chk1	9.00
9	PLK1	Serine/threonine-protein kinase PLK1	8.00
10	TTK	Dual specificity protein kinase TTK	8.00

### Molecular docking validation of 6-MDS and 10 hub genes

**Fig. 4:**
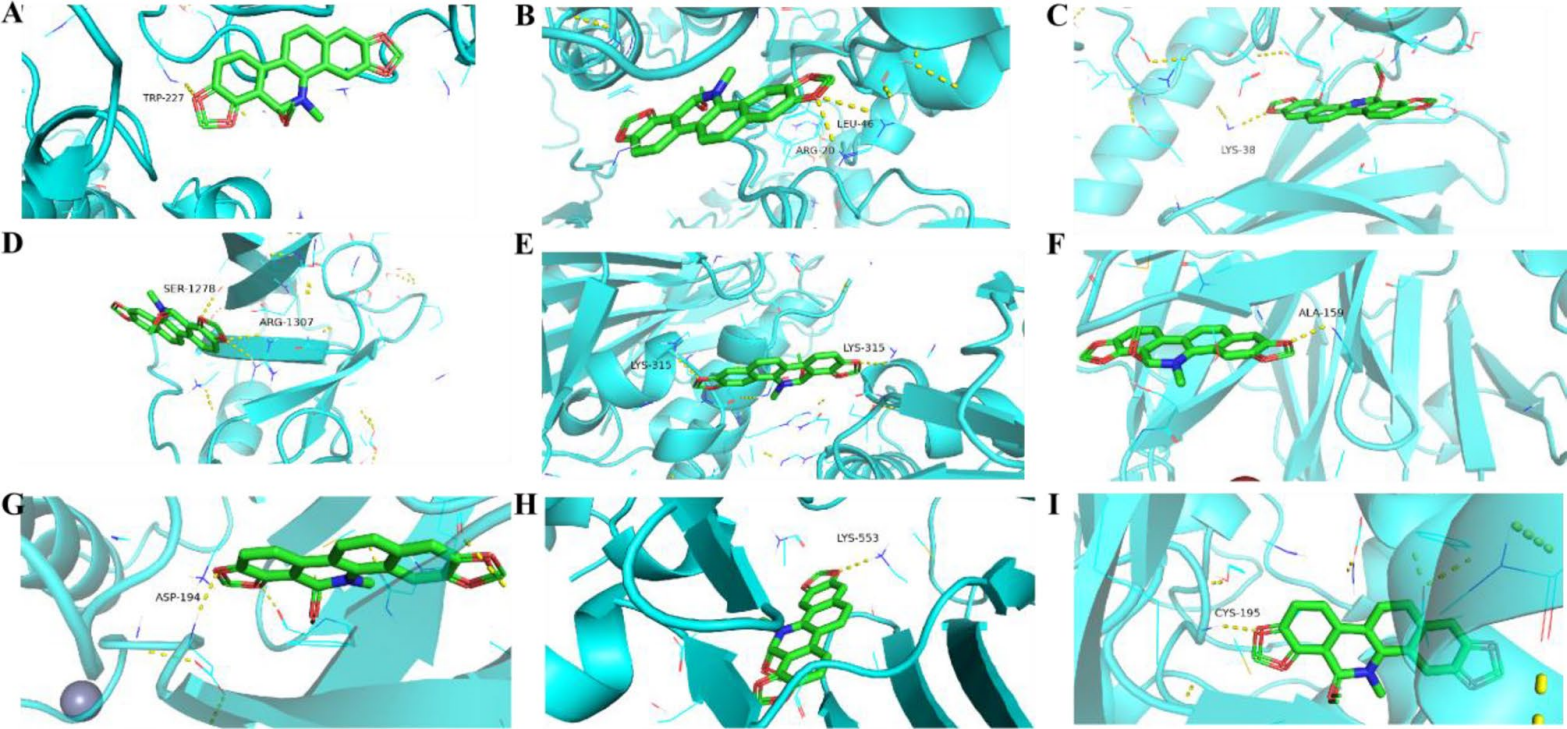
Molecular docking pattern of 6-MDS and core target protein. **A** 6-MDS-CCNA2; **B** 6-MDS-CDK1; **C** 6-MDS-CHEK1; **D**6-MDS-ERBB2; **E** 6-MDS-KIF11; **F** 6-MDS-MMP9; **G** 6-MDS-PLK1; **H** 6-MDS-TTK; **I** 6-MDS-TYMS

In order to confirm the possibility of these 10 core genes as key targets for 6-MDS treatment of LUAD, AutoDockTools-1.5.6 software was used to perform virtual molecular docking between 6-MDS and these 10 hub genes. The PDB file of the target protein is downloaded and the details of the protein are collected from the PDB database (Table 2). The parameters of the docking box are collected (Table 3). The resulting binding energy, amino acid residues, hydrogen bonds and Kd value were shown in Table 4, the outcomes of molecular docking were visualized in Fig. 4. Notably, the binding energies between 6-MDS and the hub proteins were all higher than − 6 kcal/Mol except for AURKB, suggesting robust affinity between 6-MDS and 9 of the targets. Remarkably, the binding energy of 6-MDS with PLK1 was − 11.90 kcal/mol, exhibiting the lowest docking energy among the 10 hub genes. In summary, the results indicated that 6-MDS binds strongly to core target proteins, and 6-MDS may exert anti-cancer effects by binding to core target proteins.

**Table 2:** Details of the protein targets in the PDB database

Targets	PDB ID	Method	Resolution (Å)	*R*-Value free	*R*-Value work	*R*-Value observed
MMP9	1ITV	X-RAY DIFFRACTION	1.95 Å	0.275	0.229	0.232
CDK1	4YC6	X-RAY DIFFRACTION	2.60 Å	0.267	0.225	0.227
TYMS	1HZW	X-RAY DIFFRACTION	2.00 Å	0.239	0.206	\
CCNA2	1E9H	X-RAY DIFFRACTION	2.50 Å	0.273	0.225	0.225
ERBB2	1MFG	X-RAY DIFFRACTION	1.25 Å	0.165	0.128	0.130
KIF11	2G1Q	X-RAY DIFFRACTION	2.51 Å	0.279	0.234	0.234
PLK1	8BJT	X-RAY DIFFRACTION	2.19 Å	0.255	0.200	0.203
TTK	3CEK	X-RAY DIFFRACTION	2.30 Å	0.250	0.184	0.188
CHEK1	6FCK	X-RAY DIFFRACTION	1.90 Å	0.214	0.174	0.176
AURKB	4AF3	X-RAY DIFFRACTION	2.75 Å	0.264	0.205	0.208

**Table 3:** Grid docking parameters in molecular docking

Target name	PDB ID	Spacing (angstrom)	X center	Y Center	Z center
MMP9	1ITV	1.000	-27.273	-40.514	-12.717
CDK1	4YC6	1.000	-14.325	12.348	-22.280
TYMS	1HZW	1.000	8.628	75.574	10.988
CCNA2	1E9H	0.981	11.114	27.215	90.265
ERBB2	1MFG	0.375	8.805	5.734	26.156
KIF11	2G1Q	0.964	29.537	5.655	79.451
PLK1	8BJT	0.886	-26.980	22.791	-9.754
TTK	3CEK	0.786	-4.222	16.249	34.182
CHEK1	6FCK	0.847	14.054	0.245	35.894
AURKB	4AF3	0.983	15.553	-16.602	-3.528

**Table 4:** Basic information on the molecular docking of 6-MDS and target proteins

Molecular name	Targets	PDB ID	Residue involved in H bonding	Binding energy (kcal/Mol)	Kd(mol/L)
6-MDS	MMP9	1ITV	ALA-159	-8.30	8.12 × 10 − 7
6-MDS	CDK1	4YC6	ARG-20,LEU-46	-8.70	4.13 × 10 − 7
6-MDS	TYMS	1HZW	CYS-195	-9.40	1.27 × 10 − 7
6-MDS	CCNA2	1E9H	TRP-227	-8.00	1.35 × 10 − 6
6-MDS	ERBB2	1MFG	SER-1278,ARG-1307	-6.90	8.64 × 10 − 6
6-MDS	KIF11	2G1Q	LYS-315	-8.00	1.35 × 10 − 6
6-MDS	PLK1	8BJT	ASP-194	-11.90	1.85 × 10 − 9
6-MDS	TTK	3CEK	LYS-553	-8.70	4.13 × 10 − 7
6-MDS	CHEK1	6FCK	LYS-38	-9.40	1.27 × 10 − 7
6-MDS	AURKB	4AF3	/	/	/

### External validation of the 9 hub genes

#### The mRNA expression levels of 9 hub genes

**Fig. 5:**
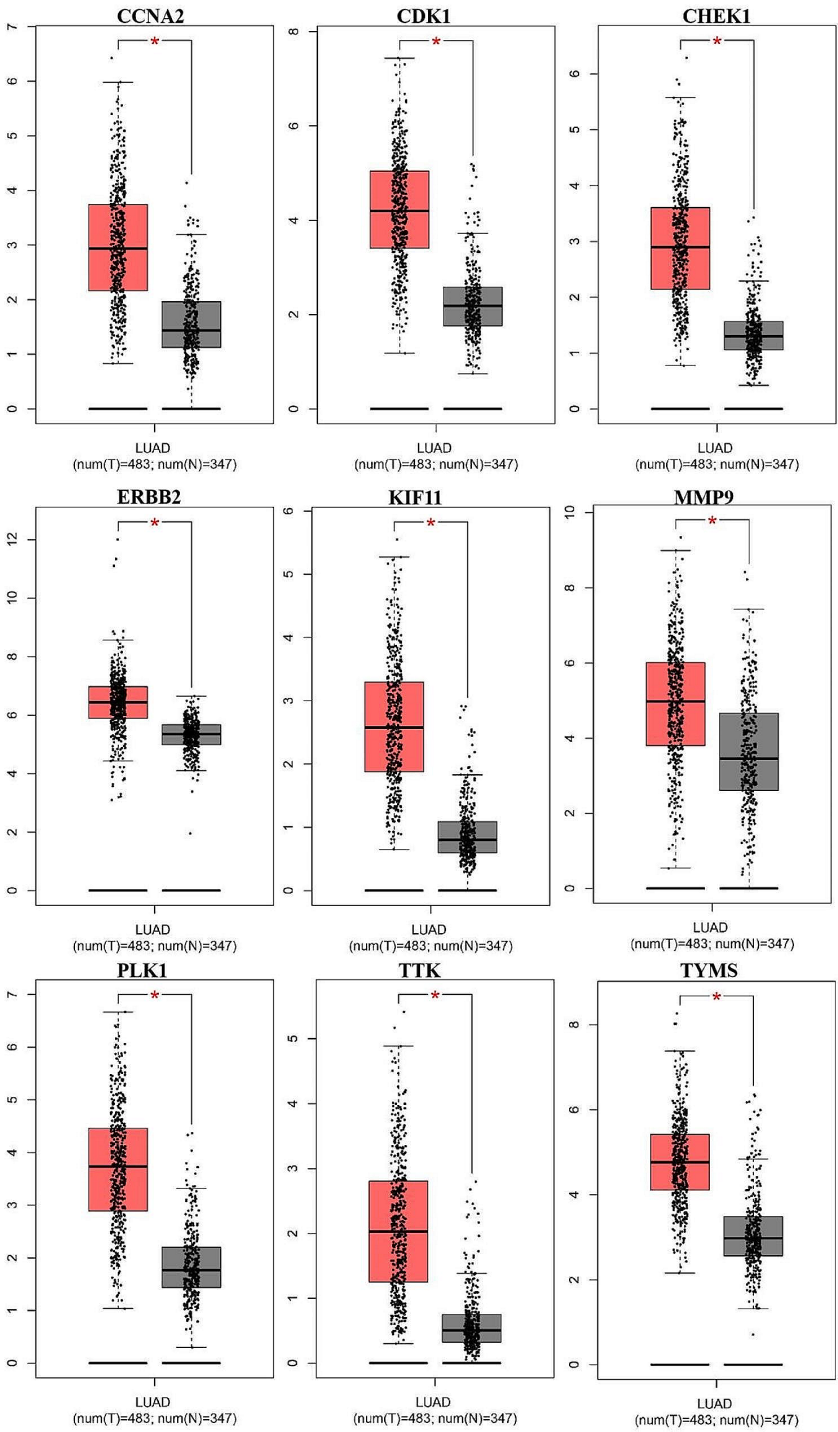
Box plot of hub gene mRNA expression levels in the GEPIA database. Red represents tumor tissues and gray represents normal tissues

**Fig. 6:**
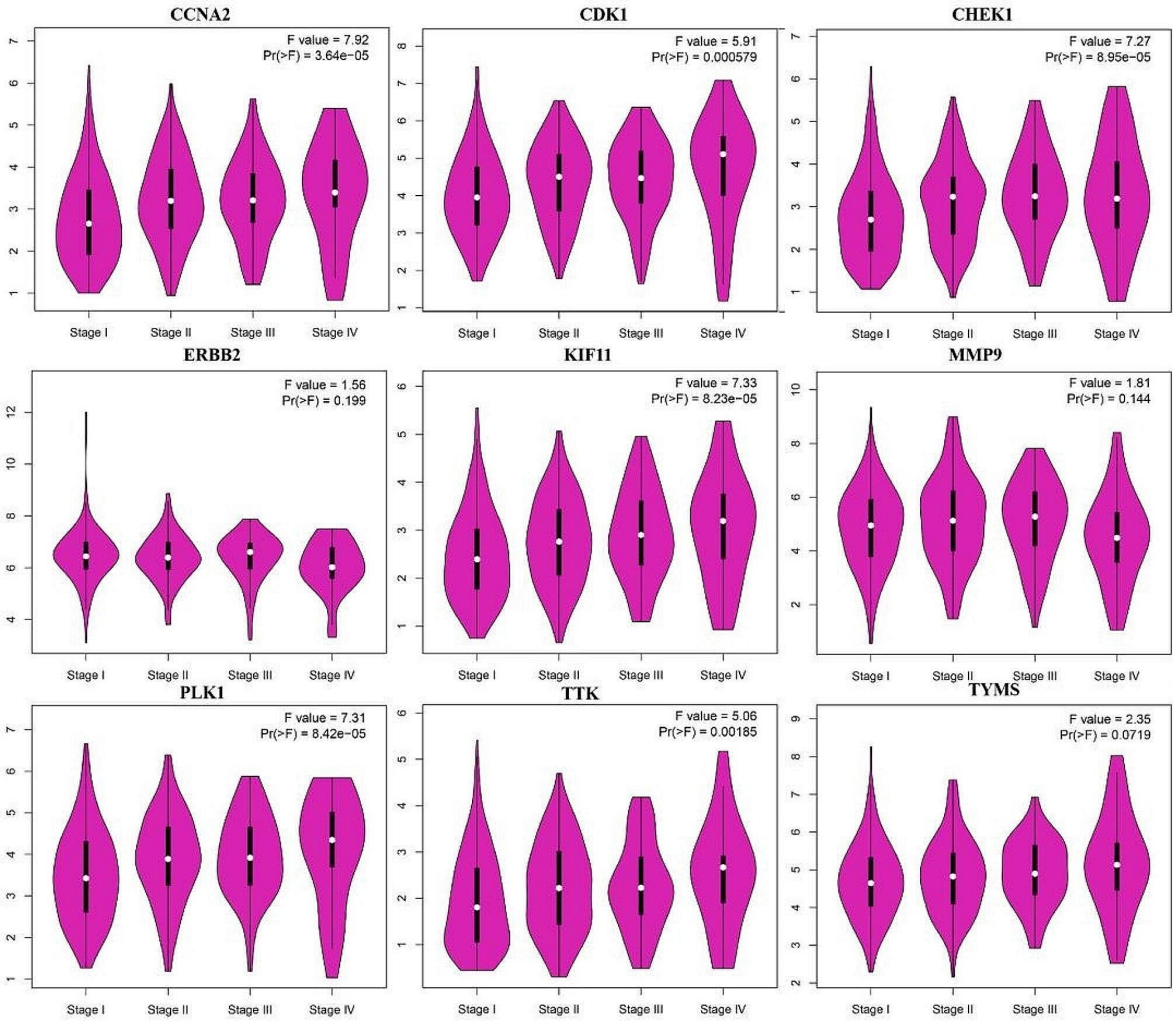
Stage diagram of hub gene mRNA expression levels and pathological stages in the GEPIA database

Since the AURKB showed no Binding energy with 6-MDS, it was excluded for further validation. The results from the GEPIA showed the expression level of the other 9 hub genes was much higher in cancer tissues than in normal tissues for LUAD (Fig. 5). Besides, the expression level of CDK1, CCNA2, CHEK1, KIF11, PLK1 and TTK was significantly elevated with cancer progression in LUAD (Fig. 6).

#### The protein expression levels of 9 hub genes

**Fig. 7:**
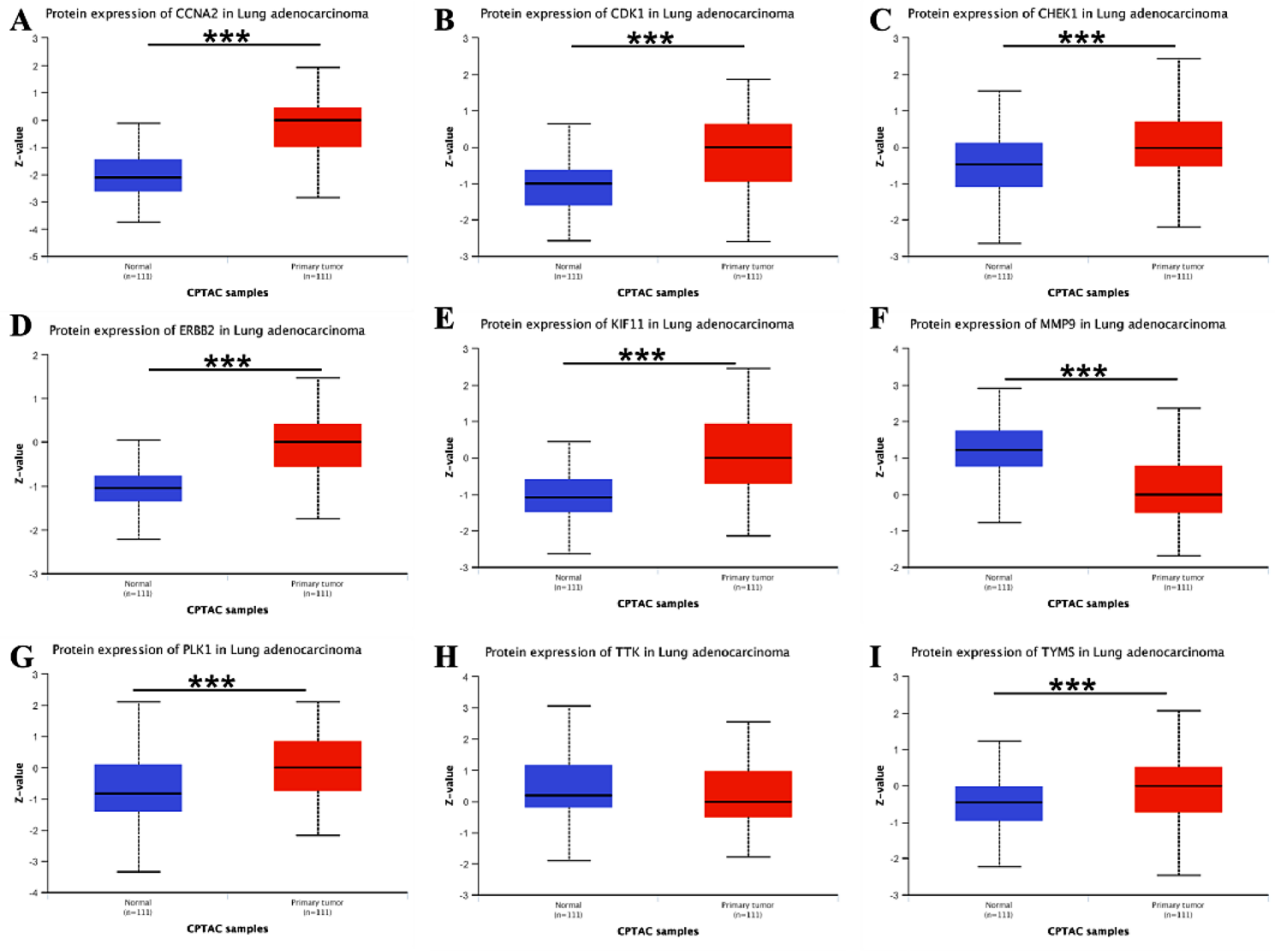
Box plot of hub protein expression levels in the UALCAN database. Red represents tumor tissues and Blue represents normal tissues. **A** CCNA2; **B** CDK1; **C **CHEK1; **D **ERBB2; **E** KIF11; **F** MMP9; **G** PLK1; **H** TTK; **I** TYMS

Consistent with the gene expression pattern, the protein expression levels of CDK1, TYMS, CCNA2, ERBB2, CHEK1, KIF11and PLK1 in LUAD tissues was significantly up-regulated compared with normal lung tissues (Fig. 7). However, the protein expression level of MMP9 was significantly down-regulated in LUAD tissue compared with normal lung tissues, which was contrary to its gene expression pattern and needs to be verified through subsequent experiments.

#### Overall survival analysis of 9 hub genes

**Fig. 8:**
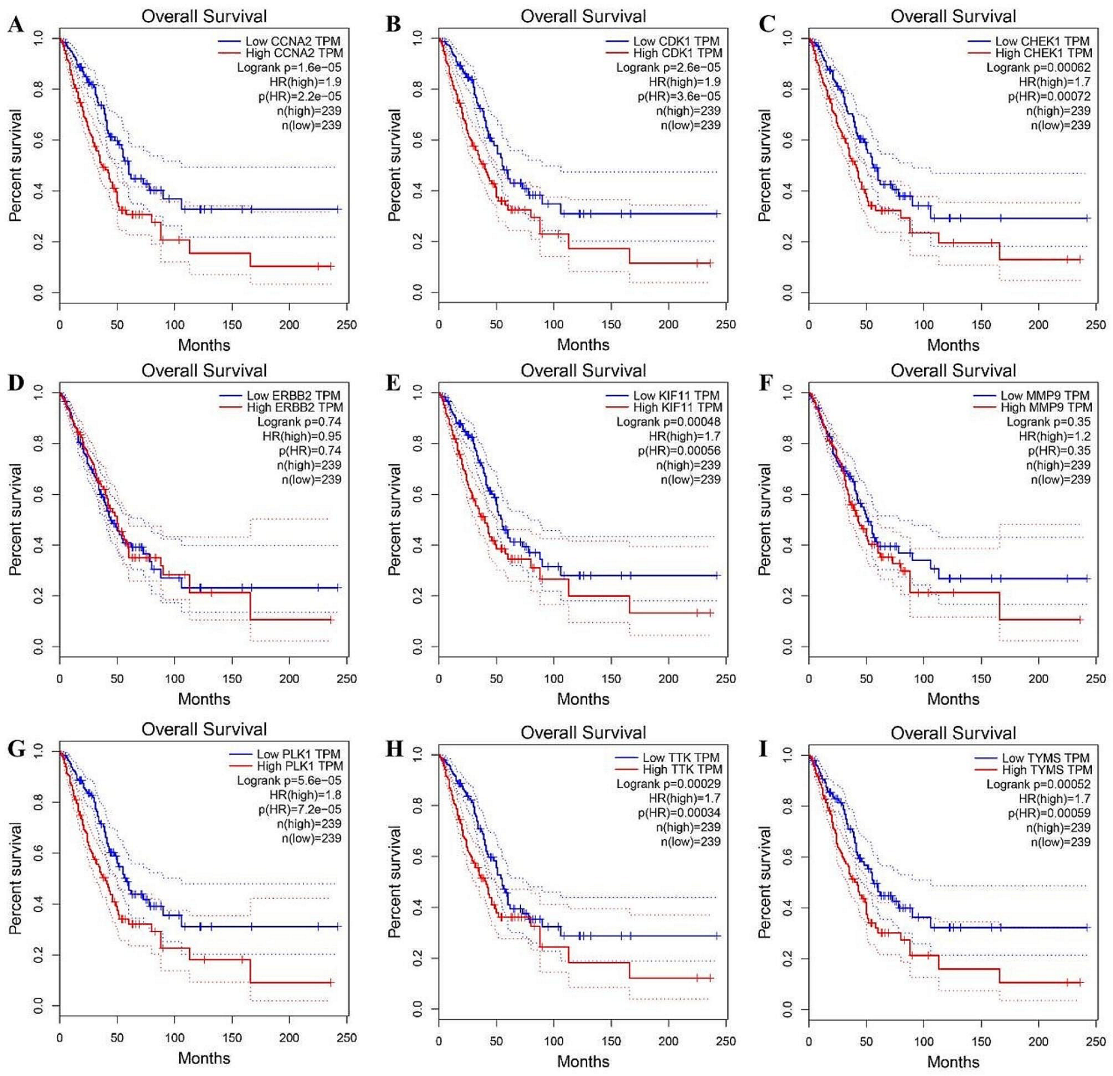
Overall survival analysis of hub gene in the GEPIA database. **A** CCNA2; **B** CDK1; **C **CHEK1; **D **ERBB2; **E** KIF11; **F** MMP9; **G** PLK1; **H** TTK; **I **TYMS

As for overall survival analysis of 9 hub genes, the results indicated that high expression of eight out of ten hub genes, including CDK1, TYMS, CCNA2, CHEK1, KIF11, PLK1 and TTK were significantly associated with the OS of LUAD patients (Fig. 8).

#### Analysis of promotor methylation level of hub genes

**Fig. 9:**
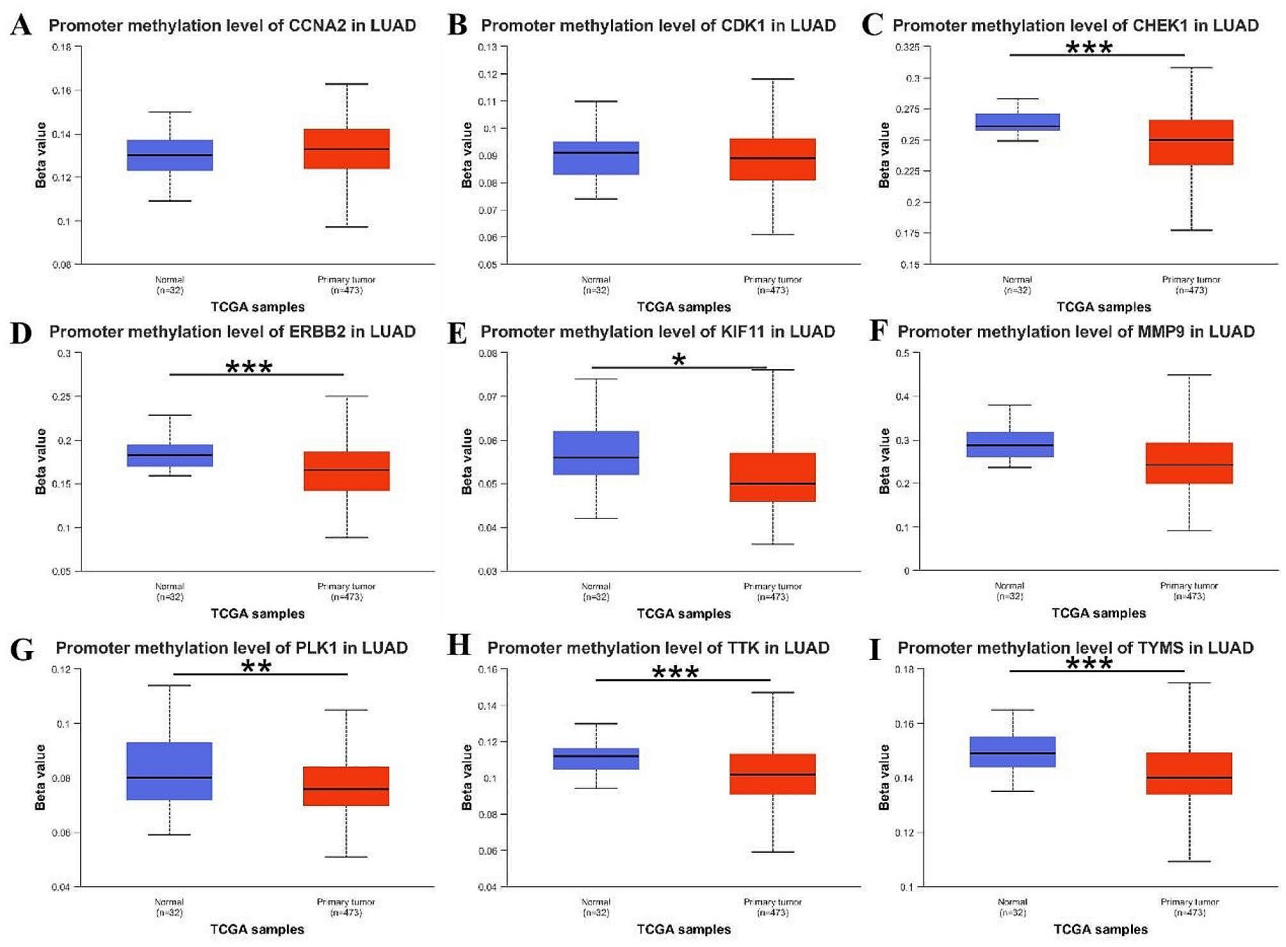
Promotor methylation level of hub genes in the UALCAN database. **A** CCNA2; **B** CDK1; **C** CHEK1; **D** ERBB2; **E** KIF11; **F** MMP9; **G** PLK1; **H** TTK; **I** TYMS

The dysregulation of DNA methylation has been implicated in the development of cancer, and used for cancer diagnosis and therapy [47, 48]. Therefore, the DNA methylation level of the 9 hub genes were compared between normal and cancer tissues by using the UALCAN databases. The result revealed that the promotor methylation level of TYMS, ERBB2, CHEK1, KIF11, PLK1 and TTK were significantly decreased in LUAD tissues compared with that in normal tissues (Fig. 9).

#### Immune cell infiltration of hub genes

**Fig. 10:**
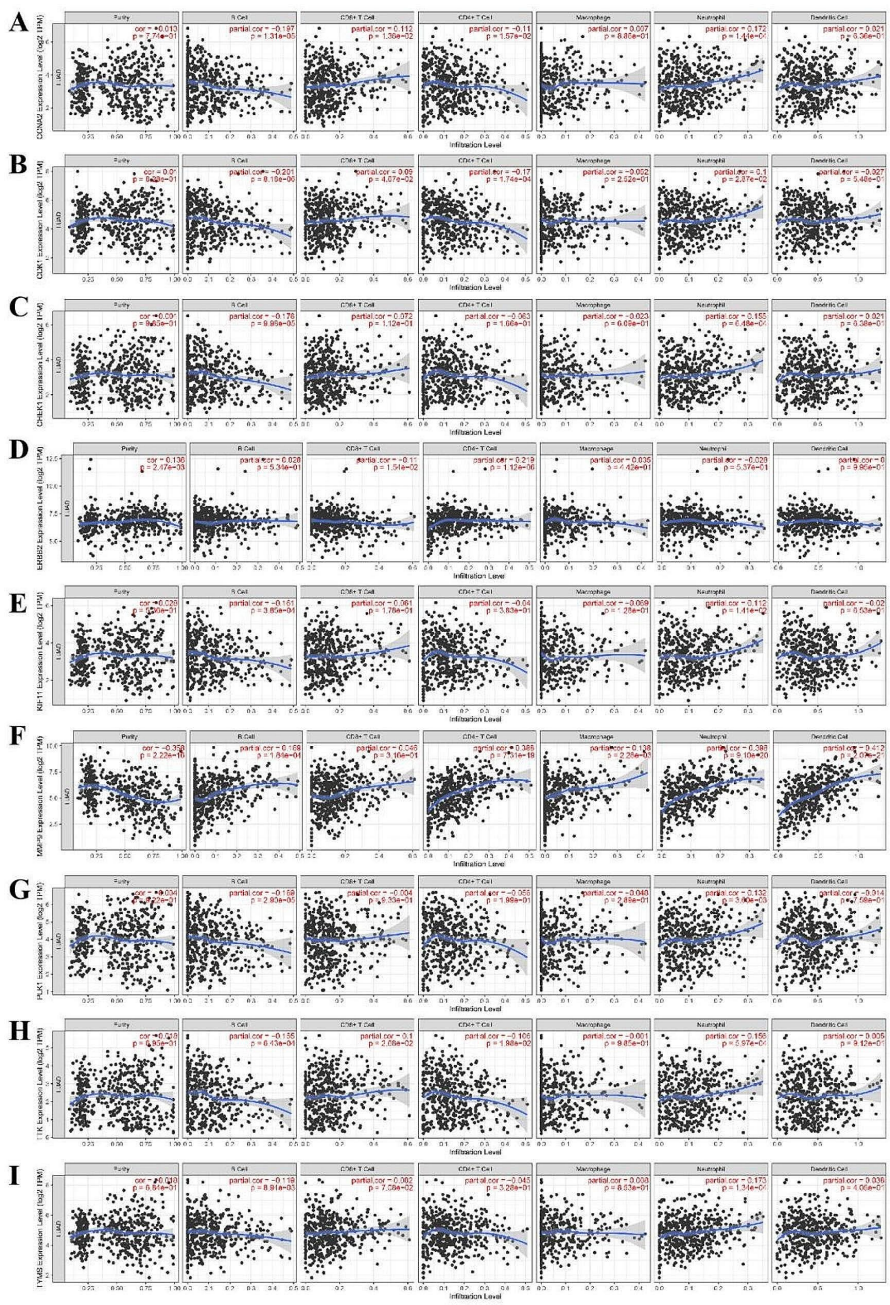
Immune cell infiltration of core targets in the Timer database. **A** CCNA2; **B** CDK1; **C** CHEK1; **D** ERBB2; **E** KIF11; **F** MMP9; **G** PLK1; **H** TTK; **I** TYMS

The 9 hub genes effects on the immunological milieu of tumors were investigated by assessing the association of its expression with the degree of immune cell infiltration. As is shown in Fig. 10, the expression of CCNA2, CDK1 and TTK were positively correlated with the infiltration of CD8 + T cells and neutrophils, while it was negatively correlated with the infiltration of B cell and CD4 + T cell. The expression of CHEK1 and KIF11 were positively correlated with infiltration of neutrophils, while negatively correlated with B cells. As for ERBB2, its expression level was negatively correlated with the infiltration of CD8 + T cell and positively correlated with the infiltration of B cell. The expression of PLK1 and TYMS were positively correlated with infiltration of neutrophils cells, while negatively correlated with the infiltration of B cell. The expression level of MMP9 was negatively correlated with purity, while positively related to the infiltration of B cell, CD4 + T cell, Macrophage, neutrophil and Dendritic cell.

#### Correlation of hub genes expression with immune subtypes

**Fig. 11:**
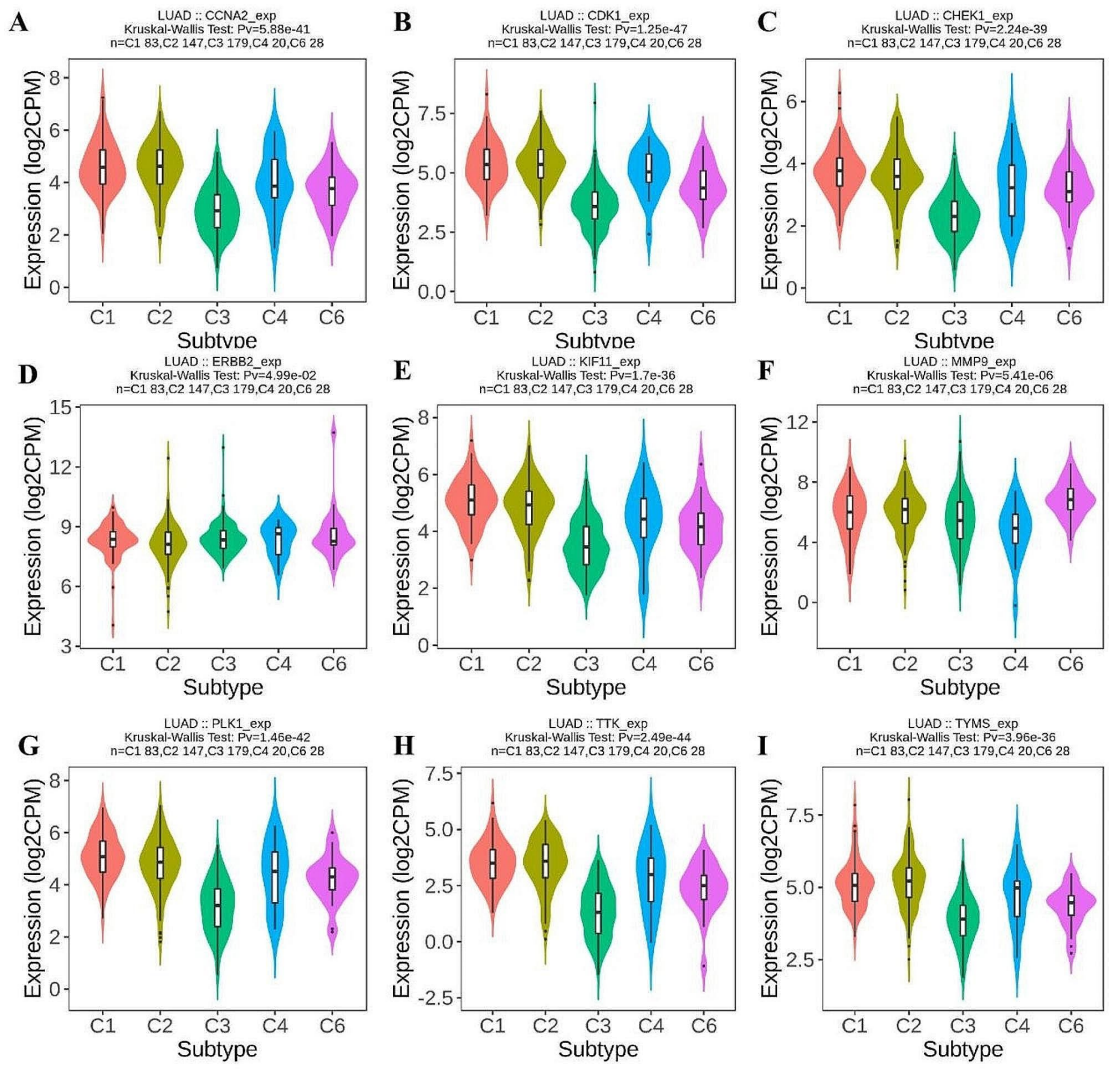
Correlation of hub genes expression with immune subtypes in the TISIDB database. **A** CCNA2; **B** CDK1; **C** CHEK1; **D** ERBB2; **E** KIF11; **F** MMP9; **G** PLK1; **H** TTK; **I** TYMS

The TISIDB online tool was ultilized to analyze the relationship between the 9 hub genes expression and LUAD immune subtypes. The results obtained from the TISIDB indicated that the expression level of the 9 hub genes was significantly associated with different immune subtypes in LUAD (Fig. 11).

### 6-MDS down-regulated the mRNA expression level of target genes

**Fig. 12:**
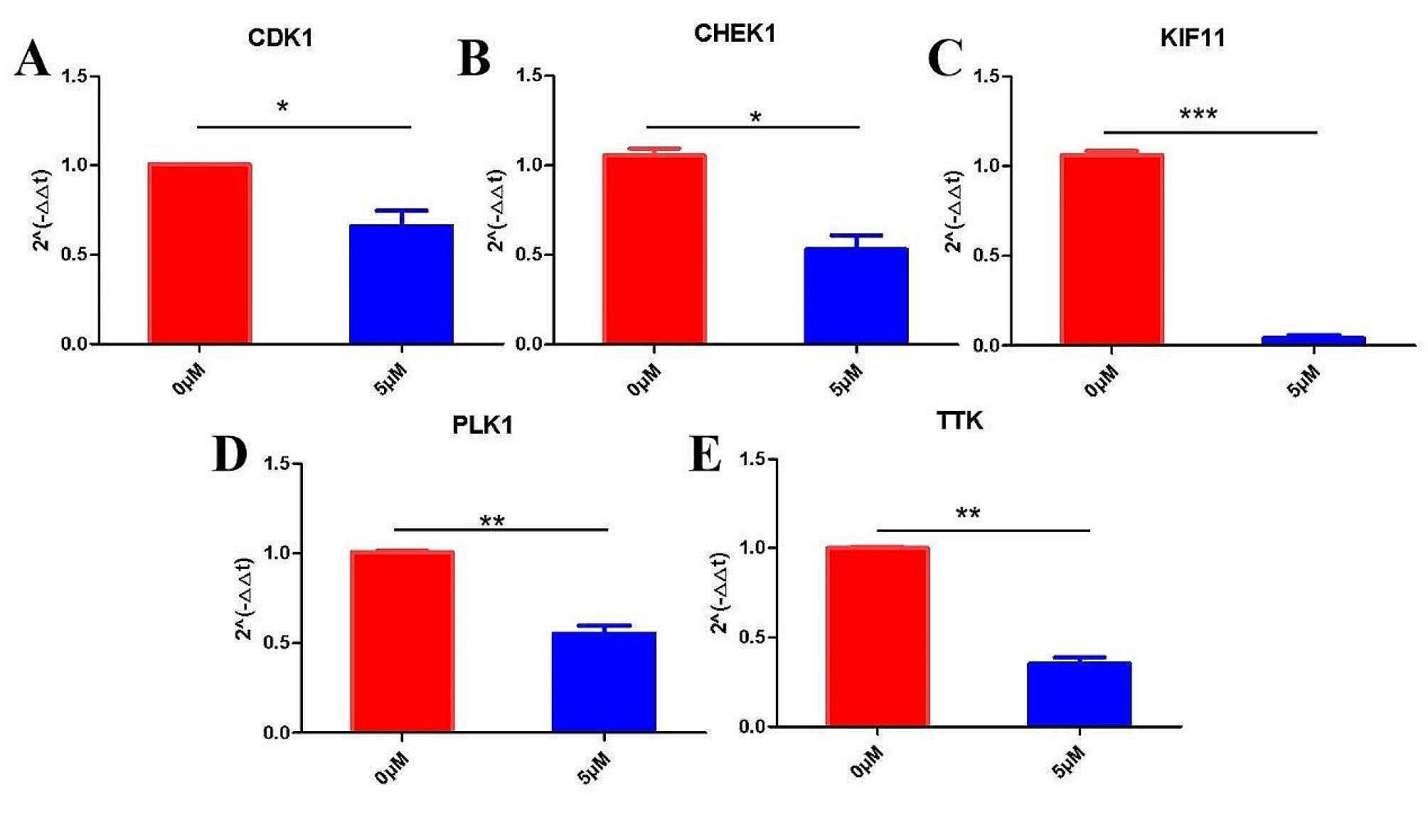
Effect of 6-MDS on the expressions of CDK1, CHEK1, KIF11, PLK1, and TTK were detected using qRT-PCR assay **(A-E)**

Since CDK1, CHEK1, KIF11, PLK1 and TTK was significantly elevated with cancer progression in LUAD and exhibited excellent binding performance with 6-MDS, they were chosen for further validation. The results of qPCR indicated that after the intervention of 5 µM 6-MDS for 24 h, the mRNA expression of CDK1, CHEK1, KIF11, PLK1 and TTK decreased significantly compared with the untreated group (*P* < 0.05). (Fig. 12A-E).

## Discussion

Lung cancer is the leading cause of mortality from cancer worldwide. Lung adenocarcinoma (LUAD) is a type of non-small cell lung cancer (NSCLC) with highest prevalence. Despite advancements in targeted therapy and immunotherapy, the overall survival of LUAD patients remain discouraging due to the metastases [49]. Natural products have recently garnered significant interest owing to their potential anti-cancer effects, which could pave the way for the development of innovative medications. 6-MDS is a natural benzophenanthridine alkaloid [50] which has shown promising anti-cancer effects. However, whether 6-MDS exhibits anti-cancer properties on LUAD and the underlying pharmacological mechanism needs further study.

In recent decades, the integration of network-based pharmacology and computer-assisted drug design technology has gained traction in uncovering the intricate workings of drugs, emerging as a potent approach in pharmaceutical investigations [51–55]. Among them, network pharmacology enables the anticipation of disease targets impacted by drugs, while molecular docking facilitates the examination of drug-gene interactions. In this study, a combination of bio-information analysis and network pharmacology, as well as molecular docking, were used to simulate the possible mechanisms of 6-MDS treatment for LUAD. Through network pharmacology, we identified 10 core genes that may be potential targets for 6-MDS treatment of LUAD. KEGG enrichment analysis revealed that these genes were mainly enriched in cell cycle pathway. Cell cycle regulation is orchestrated by a complex network of interactions between proteins, enzymes, cytokines, and cell cycle signaling pathways, and is vital for cell proliferation, growth, and repair. It is well-established that the occurrence, development, and metastasis of tumors are intricately linked with the regulation of the cell cycle [56]. These finding proposed that 6-MDS may mainly inhibit the occurrence of LUAD by affecting the cell cycle.

The molecular docking analysis unveiled that nine targets had good binding performance with 6-MDS. Notably, the binding energy of 6-MDS with PLK1 was − 11.90 kcal/mol, which had the lowest docking energy mong the targets examined. Besides, the binding energy of CDK1, CHEK1, KIF11 and TTK were all lower than − 8.7 kcal/mol, indicating strong binding capabilities. In addition, the expression level of CDK1, CHEK1, KIF11, PLK1 and TTK was significantly elevated with cancer progression in LUAD. And they also demonstrated a significant correlation with the OS of LUAD patients.

Polo-like kinase 1 (PLK1) is crucial for the normal progression of mitosis. The significant upregulation of PLK1 has been found in various human cancers and is significantly associated with poor prognosis in various cancers. Many studies have showed that inhibition of PLK1 could lead to death of cancer cells by interfering with multiple stages of mitosis. In the case of LUAD, PLK1 was found to be highly expressed in LUAD and was positively associated with advanced disease staging and poor survival outcomes. Also, PLK1 plays a critical role in LUAD progression by regulating necroptosis and immune infiltration, and may serve as a potential therapeutic target for immunotherapy [57, 58]. Cyclin dependent kinases (CDKs) are serine/threonine kinases that are proposed as promising candidate targets for cancer treatment [59]. Deregulation of CDK1 has been shown to be closely associated with tumorigenesis. CDK1 activation plays a critical role in a wide range of cancer types; and CDK1 phosphorylation of its many substrates greatly influences their function in tumorigenesis. These proteins complexed with cyclins play a critical role in cell cycle progression. CDK1 is a potential prognostic biomarker and target for lung cancer; CDK1 activity is critical for JAK/STAT3 signaling activation, and the inhibition of CDK1 can suppress lung cancer. In addition, an in vitro study of LUAD cells showed that reduced CDK1 activity led to cell cycle arrest and promotion of apoptosis in LUAD [60]. Hence, CDK1 may be used as potential biomarkers and therapeutic targets for LUAD [61].Checkpoint kinase 1 (CHEK1, also known as CHK1) is a conserved serine/threonine kinase that plays an important role in replication fork stability and DNA damage response [62]. In a study of TP53 mutant NSCLC tumor cells, it was found that inhibiting the expression of CHEK1 can significantly enhance the sensitivity of tumor cells to chemotherapy [63, 64]. In addition, promoter methylation, amplification, and miRNA regulation in patients with lung adenocarcinoma may lead to the upregulation of the CHEK1 gene, which may be a marker for predicting the survival rate of patients with lung adenocarcinoma [65]. The motor protein superfamily consists of 45 family members, among which KIF11 plays a role as a motor protein in mitosis. KIF11 is essential for LUAD cell proliferation and metastasis, and it may serve as an independent prognostic factor as well as a promising therapeutic target for LUAD patients [66]. TTK, also known as Monopolar spindle1 (Mps1), is the crucial modulator of the spindle assembly checkpoint, which is responsible for ensuring chromosomal separation. At present, some studies have found that TTK may be related to the occurrence and development of lung cancer. Zheng et al. showed that the expression of TTK was higher in lung adenocarcinoma and squamous cell carcinoma than in normal lung tissue, which was related to the poor prognosis of patients with lung adenocarcinoma [67]. When TTK was knocked out in A549 cell, cell proliferation, migration and tumorigenesis were inhibited [68]. Therefore, TTK may be a promising prognostic biomarker for LUAD and is worthy of further investigation [69]. Furthermore, qPCR resulted indicated that after the intervention of 5 µM 6-MDS for 24 h, the mRNA expression of CDK1, CHEK1, KIF11, PLK1 and TTK decreased significantly compared with the untreated group. In conclusion, the 5 key targets, which had good binding force with 6-MDS may play an important role in cancer progression, which preliminarily confirmed the possibility of 6-MDS against LUAD at the molecular level.

Our present study has certain limitations. Firstly, this study reveals possible targets and pathways of the impact of 6-MDS, but our understanding of the exact mechanism by which 6-MDS exerts its anti-tumor properties is still quite limited. Deeper exploration into the downstream signaling pathways and molecular mechanisms that drive the activity of 6-MDS could provide valuable insights into the discovery. Secondly, it is not hard to argue that more accurate experiments should be carried out, such as surface plasmon resonance (SPR) or Cellular Thermal Shift Assay (CETSA) to confirm that 6-MDS does indeed treat lung adenocarcinoma through the above mentioned targets. Furthermore, the incorporation of animal models or clinical trials is imperative to establish stronger evidence regarding the efficacy and safety of 6-MDS for LUAD treatment. Nevertheless, the current limitations in resources and time have confined us to performing only fundamental experiments. Thirdly, it should be noted that the limited water solubility and fast metabolism of 6-MDS may hinder its medical applications, despite its encouraging anti-cancer properties. Therefore, innovative approaches like nanocarriers need to be developed and explored to enhance the bioavailability of 6-MDS. The next phase of our research will focus on this particular area.

## Conclusions

To sum up, a comprehensive evaluation of 6-MDS was performed for revealing its potential mechanism for the treatment of LUAD through network pharmacology, molecular docking and experimental validation. The results of this study suggested that 6-MDS might be a candidate used for treating LUAD. More studies are required to validate its anti-cancer effect and explore the underlying mechanisms.

### Electronic supplementary material

Below is the link to the electronic supplementary material.

Supplementary Material 1

## Data Availability

The original contributions presented in the study are included in the article/supplementary material. All other relevant data are available from the corresponding authors upon reasonable request.
